# Can Transcutaneous CO_2_ Tension Be Used to Calculate Ventilatory Dead Space? A Pilot Study

**DOI:** 10.1155/2016/9874150

**Published:** 2016-09-05

**Authors:** Pradeep H. Lakshminarayana, Adiba A. Geeti, Umer M. Darr, David A. Kaufman

**Affiliations:** ^1^Section of Pulmonary, Critical Care and Sleep Medicine, Bridgeport Hospital, Yale New Haven Health, 267 Grant Street, Bridgeport, CT 06610, USA; ^2^Yale Cardiac Surgery, Bridgeport Hospital, 267 Grant Street, Bridgeport, CT 06610, USA

## Abstract

Dead space fraction (*V*_d_/*V*_t_) measurement performed using volumetric capnography requires arterial blood gas (ABG) sampling to estimate the partial pressure of carbon dioxide (P_a_CO_2_). In recent years, transcutaneous capnography (P_tc_CO_2_) has emerged as a noninvasive method of estimating P_a_CO_2_. We hypothesized that P_tc_CO_2 _can be used as a substitute for P_a_CO_2_ in the calculation of *V*_d_/*V*_t_. In this prospective pilot comparison study, 30 consecutive postcardiac surgery mechanically ventilated patients had *V*_d_/*V*_t_ calculated separately using volumetric capnography by substituting P_tc_CO_2_ for P_a_CO_2_. The mean *V*_d_/*V*_t_ calculated using P_a_CO_2_ and P_tc_CO_2_ was 0.48 ± 0.09 and 0.53 ± 0.08, respectively, with a strong positive correlation between the two methods of calculation (Pearson's correlation = 0.87, *p* < 0.05). Bland-Altman analysis showed a mean difference of −0.05 (95% CI: −0.01 to −0.09) between the two methods. P_tc_CO_2_ measurements can provide a noninvasive means to measure *V*_d_/*V*_t_, thus accessing important physiologic information and prognostic assessment in patients receiving mechanical ventilation.

## 1. Introduction

The ventilatory dead space fraction (*V*_d_/*V*_t_) is defined as the portion of tidal volume that does not participate in gas exchange because it does not reach perfused lung units. Clinically, *V*_d_/*V*_t_ may be measured using volumetric capnography and the Enghoff modification of the Bohr equation, which states

(1) $$\begin{array}{c} \frac{V_{d}}{V_{t}}=\frac{\left(P_{a}{CO}_{2}-P_{e}{CO}_{2}\right)}{\left(P_{a}{CO}_{2}\right)}, \end{array}$$

where P_a_CO_2_ is the partial pressure of carbon dioxide in the arterial blood and P_e_CO_2_ is the partial pressure of carbon dioxide in exhaled air [1]. *V*_d_/*V*_t_ may increase in a variety of pathological situations, especially conditions with obstruction of the pulmonary vasculature [2]. Measuring *V*_d_/*V*_t_ requires arterial blood gas (ABG) sampling to measure P_a_CO_2_ [3]. Transcutaneous capnography (P_tc_CO_2_) has emerged as a noninvasive method of estimating P_a_CO_2_ [4]. The objective of our study was to evaluate the relationship between P_tc_CO_2 _and P_a_CO_2_ in calculating *V*_d_/*V*_t_. We hypothesized that P_tc_CO_2 _can be used as a substitute for P_a_CO_2_ in the calculation of *V*_d_/*V*_t_.

## 2. Methods

This was a prospective pilot comparison study conducted at a single 383-bed urban, community-teaching hospital. We recruited thirty consecutive adult patients admitted to the Surgical Intensive Care Unit (SICU) after cardiac surgery from May 1, 2014, until December 1, 2014. We excluded patients who were extubated within 4 hours of arrival to the SICU. The Bridgeport Hospital Institutional Review Board (IRB) approved this study.

Volumetric capnography was performed within 12 hours of patient arrival to the SICU. A CAPNOSTAT® CO_2_ sensor was attached between the subject's endotracheal tube and the Y-piece of the ventilator circuit to obtain breath-by-breath volumetric capnography using NICO_2_ volumetric capnography (Philips-Respironics®, Wallingford, CT). We measured P_tc_CO_2 _using the Tosca 500 (Radiometer®, Copenhagen, Denmark), according to the manufacturer's instructions. A fully automated calibration of the transcutaneous CO_2_ sensor (Tosca sensor 92) containing a Stow-Severinghaus type electrode was performed using CAL-Gas (Radiometer), which contained 12% O_2_, 7% CO_2_, and 81% N_2 _prior to each monitoring period and every time the membrane of the transcutaneous sensor was changed (every 14 days). First, the sensor probe and the subject's earlobe were cleaned with alcohol and dried prior to each measurement. Then, the sensor was placed on the earlobe after placing a drop of contact solution provided by the manufacturer and secured by means of an adhesive clip. No P_tc_CO_2_ values were collected until the ear lobe reached an appropriate temperature of 42°C and stable signals for both P_tc_CO_2_ and P_e_CO_2_ were achieved after at least 15 minutes. The P_tc_CO_2_ was recorded at the same time an arterial blood sample was collected from an existing radial arterial line, placed on ice, and sent immediately for standard blood gas analysis. P_a_CO_2_ was measured using the arterial blood gas sample analyzed with Cobas b221 blood gas analyzer (Roche®). Turnaround time for the result was under 10 minutes for all samples. We calculated *V*_d_/*V*_t_ using both simultaneously measured values for P_a_CO_2_ and P_tc_CO_2_ using NICO_2_ volumetric capnography according to the manufacturer's instructions. In this study, we used single measurements of P_e_CO_2_, P_a_CO_2_, and P_tc_CO_2_ to calculate *V*_d_/*V*_t_, with all values obtained simultaneously. The *V*_d_/*V*_t_ calculated using P_a_CO_2_ was considered the reference standard [5].

### 2.1. Statistical Analysis

Statistical analysis was performed using SPSS version 9.0. Results from descriptive statistics are presented as mean ± standard deviation (SD). Linear regression and Bland-Altman analyses were performed to compare the values of *V*_d_/*V*_t_ using P_tc_CO_2 _and P_a_CO_2_ measurements.

## 3. Results

**Figure 1:**
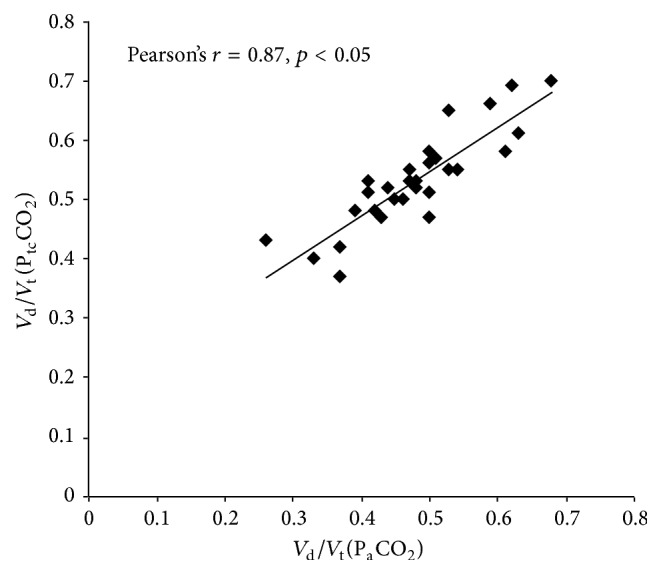
Scatter plot of *V*_d_/*V*_t_ using P_a_CO_2_ against *V*_d_/*V*_t_ using P_tc_CO_2_.

**Figure 2:**
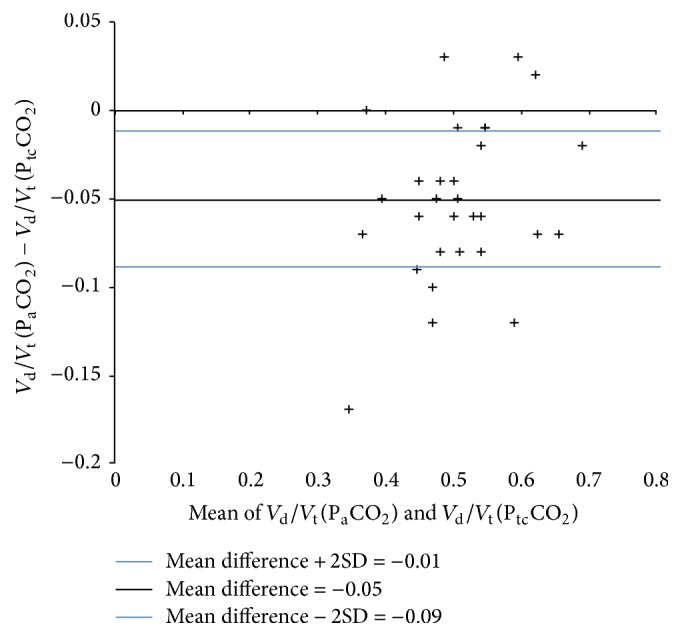
Modified Bland-Altman plot of *V*_d_/*V*_t_(P_a_CO_2_) − *V*_d_/*V*_t_(P_tc_CO_2_) against mean of *V*_d_/*V*_t_(P_a_CO_2_) and *V*_d_/*V*_t_(P_tc_CO_2_).

30 consecutive postcardiac surgery patients were included and 30 separate volumetric capnography measurements were obtained. Statistical description of the study population can be found in Table 1. 24 patients were males, mean age was 68.25 ± 6.36 years, and the mean Acute Physiology and Chronic Health Evaluation (APACHE) II score was 14.5 ± 3.92. The mean *V*_d_/*V*_t_ calculated using P_a_CO_2_ and P_tc_CO_2_ was 0.48 ± 0.09 and 0.53 ± 0.08, respectively, with a strong positive correlation between the two methods of calculation (Pearson's correlation = 0.87, *p* < 0.05) as shown in Figure 1. Bland-Altman analysis showed a mean difference of −0.05 (95% CI: −0.01 to −0.09) between the two methods of *V*_d_/*V*_t_ measurements as shown in Figure 2.

## 4. Discussion

Measuring *V*_d_/*V*_t_ at the bedside can be easily performed with volumetric capnography, in which both exhaled tidal volume and P_e_CO_2_ are measured. The P_e_CO_2 _is compared to P_a_CO_2_ determined from the arterial blood, and the Enghoff modification of the Bohr equation is used to calculate *V*_d_/*V*_t_ [3]. *V*_d_/*V*_t_ has been shown to be useful in identifying cardiac surgery patients with microthrombosis of the pulmonary circulation [6] and ARDS patients who have an increased risk for death [7]. It has also been proposed to help identify patients with pulmonary embolism [8] and to risk-stratify intubated patients for extubation failure [9]. A drawback of this method is that it requires sampling of the arterial blood, which can be painful and may cause complications. Thus, a noninvasive test to estimate *V*_d_/*V*_t_ might be helpful.

A transcutaneous measurement of CO_2_ is based on the principle that an increase in skin capillary blood flow facilitates diffusion of CO_2_, hence allowing its detection by a sensor located at the skin surface. The sensor is also equipped with a thermostatically controlled heater unit, which allows an increase in temperature of the skin surface. The Stove-Severinghaus type CO_2_ sensor is a potentiometric sensor combining a silver/silver chloride reference electrode and a miniature glass pH electrode. P_tc_CO_2_ is determined by a change in pH of the electrolyte solution [4, 10].

P_tc_CO_2_ is a noninvasive method of estimating P_a_CO_2_ used frequently in clinical practice. Its application has been widely noted during mechanical ventilation, anesthesia, bronchoscopy, and sleep studies [4]. Whether substituting P_tc_CO_2 _for P_a_CO_2_ yields accurate measurements of *V*_d_/*V*_t_ is unreported in the scientific literature. We performed a prospective pilot comparison to explore whether using P_tc_CO_2_ in place of P_a_CO_2_ in the Enghoff-Bohr calculation would yield accurate estimates of *V*_d_/*V*_t_. Our observations show a strong positive correlation between both methods of estimating *V*_d_/*V*_t_ with good agreement between the two techniques.

Our study has some limitations. First, the sample size is small. We consider our findings to be preliminary. They will require confirmation in a larger cohort of patients. Second, we conducted our study exclusively in patients who had undergone cardiac surgery, both emergent and elective. We chose this group because it is relatively homogeneous with regard to complicating respiratory and metabolic disorders, thereby minimizing residual confounders that might bias the results. Whether our findings can be extrapolated to other critically ill populations will require further investigation. For example, it is plausible that transcutaneous CO_2 _measurements will be less accurate in patients with circulatory failure or shock, as peripheral blood flow may be diminished. Finally, our goal was limited. We sought to test how well *V*_d_/*V*_t_ calculations using P_tc_CO_2_ measurements compared to *V*_d_/*V*_t_ calculations using P_a_CO_2_. We did not employ a reference standard measurement of *V*_d_/*V*_t_ such as mixed inert gas elimination technique, as such techniques are found only in highly specialized research centers. It is possible that unmeasured confounders that affect volumetric capnography might result in random or systematic biases that make our findings unreliable. Indeed, P_tc_CO_2_ could be influenced by advanced age, hypothermia, hypercapnia, and use of vasopressor and inotropic support. A larger observational study would be essential to determine the influence of these potential confounders on *V*_d_/*V*_t_ measurement. Nonetheless, we believe that our methods represent a real-world approach to use bedside *V*_d_/*V*_t_ measurements and that P_tc_CO_2_ may be an acceptable substitute for P_a_CO_2_ to calculate *V*_d_/*V*_t_.

Thus, we propose that substituting P_tc_CO_2 _for P_a_CO_2_ may be clinically useful and provide a noninvasive means for estimating *V*_d_/*V*_t_. Using a noninvasive CO_2_ measurement to calculate *V*_d_/*V*_t_ potentially could permit more frequent estimates of *V*_d_/*V*_t_ when multiple samples of arterial blood are unavailable or invasive procedures are not desired. For example, it is plausible that following the *V*_d_/*V*_t_ over the course of a spontaneous breathing trial could be useful at identifying patients at high risk for requiring continued mechanical ventilator support, as has been shown in pediatric patients with respiratory failure [11]. Future work incorporating noninvasive CO_2_ measurements into *V*_d_/*V*_t_ calculations should focus on whether these estimates are valid in a broader population of critically ill patients and whether *V*_d_/*V*_t_ measurements can be incorporated into treatment strategies to yield improved patient-centered outcomes.

## 5. Conclusion

P_tc_CO_2_ may be a useful substitute for P_a_CO_2_ to calculate the dead space fraction. P_tc_CO_2_ measurements can provide a noninvasive means to measure the dead space fraction, thus allowing us to obtain important physiologic information and prognostic assessment in critically ill patients on mechanical ventilation.

## Figures and Tables

**Table 1:** Patient demographic and clinical characteristics (*n* = 30).

Mean age (years) ± SD	68.25 ± 6.36
Males	24
Types of cardiac surgery	
Valve replacement	12
CABG	12
Valve + CABG	4
Aneurysm	2
Cardiopulmonary bypass status	
On bypass	26
Off bypass	4
Mean APACHE II score ± SD	14.5 ± 3.92
Patient comorbidities^1^	
Cardiovascular	28
Respiratory	6
Endocrine	8
Neurology	3
Miscellaneous^*∗*^	4
Vasopressor or inotrope support	14
Mean tidal volume on ventilator^2^ (mL) ± SD	660 ± 95.95
Mean P_a_CO_2_ (mm of Hg) ± SD	35.93 ± 5.19
Mean P_tc_CO_2_ (mm of Hg) ± SD	39.73 ± 5.82
Mean shunt fraction (*Q*_s_/*Q*_t_)^3^	0.19 ± 0.08

^1^Patients may have more than one comorbidity.

^*∗*^Miscellaneous comorbidities included cirrhosis, chronic kidney disease, HIV and malignancy.

^2^All patients maintained on volume assist control mode of mechanical ventilation.

^3^Measured only in 27 patients who had a pulmonary artery catheter.

Abbreviations: CABG: coronary artery bypass graft; APACHE: acute physiology and chronic health evaluation.
